# Association between Virulence and Triazole Tolerance in the Phytopathogenic Fungus *Mycosphaerella graminicola*

**DOI:** 10.1371/journal.pone.0059568

**Published:** 2013-03-15

**Authors:** Lina Yang, Fangluan Gao, Liping Shang, Jiasui Zhan, Bruce A. McDonald

**Affiliations:** 1 Key Lab for Biopesticide and Chemical Biology, Ministry of Education, Fujian Agriculture and Forestry University, Fuzhou, Fujian, People's Republic of China; 2 Laboratory of Plant Virology of Fujian Province, Institute of Plant Virology, Fujian Agriculture and Forestry University, Fuzhou, Fujian, People's Republic of China; 3 Institute of Integrative Biology, ETH Zurich, Zürich, Switzerland; Soonchunhyang University, Republic of Korea; Yun

## Abstract

Host resistance and synthetic antimicrobials such as fungicides are two of the main approaches used to control plant diseases in conventional agriculture. Although pathogens often evolve to overcome host resistance and antimicrobials, the majority of reports have involved qualitative host – pathogen interactions or antimicrobials targeting a single pathogen protein or metabolic pathway. Studies that consider jointly the evolution of virulence, defined as the degree of damage caused to a host by parasite infection, and antimicrobial resistance are rare. Here we compared virulence and fungicide tolerance in the fungal pathogen *Mycosphaerella graminicola* sampled from wheat fields across three continents and found a positive correlation between virulence and tolerance to a triazole fungicide. We also found that quantitative host resistance selected for higher pathogen virulence. The possible mechanisms responsible for these observations and their consequences for sustainable disease management are discussed.

## Introduction

Knowledge of the evolutionary biology of plant pathogens is needed for sustainable disease management in agricultural systems [1]. The development of host resistance through plant breeding and applications of synthetic fungicides are two major approaches used to control fungal diseases. Plants have evolved an array of chemical, structural and enzymatic defenses to protect themselves against pathogens [2], [3], [4], [5], [6]. Chemical defenses include the production of secondary metabolites that are toxic to pathogens [7], [8]. Like the fungicidal secondary metabolites produced by plants, synthetic fungicides disrupt fungal metabolism, either inhibiting development and growth or killing the fungus outright.

The widespread use of host resistance and fungicides selects for pathogen individuals or populations that can overcome the host defense systems or that are resistant to the applied fungicides. For qualitative host – pathogen interactions following the gene-for-gene model and fungicides targeting a single fungal protein, the emergence of pathogenicity (here defined as the qualitative capacity of a parasite to infect and cause disease on a host, [9]) or fungicide resistance often results from single point mutations that occur at random in pathogen populations [10], [11], [12], [13]. Under selection, these mutations increase in frequency and can spread rapidly over large areas through natural or human-mediated gene flow. When resistance is quantitative or a fungicide targets several proteins or biochemical pathways, the emergence of virulence (here defined as the degree of damage caused to a host by parasite infection, [14]) or fungicide resistance in pathogen populations is more complex and occurs more slowly, likely involving recurring cycles of mutation-selection-recombination. Natural selection increases the frequency of phenotypes with higher fitness. New mutations or recombination among the selected phenotypes will create new genetic variation for the next cycle of selection.

The majority of studies on the evolution of plant pathogens have involved qualitative host – pathogen interactions or antimicrobials targeting a single pathogen protein or metabolic pathway. Studies that jointly consider the evolution of virulence and antimicrobial resistance are limited. Yet this type of study is important to understand the emergence of infectious diseases and to devise sustainable disease management in agriculture and medicine. In this study, we used the wheat-*Mycosphaerella graminicola* system to address the interaction of the evolution of virulence and antimicrobial resistance in agricultural ecosystems. The objectives of this study were: 1) to determine whether there is an association between virulence and resistance to fungicides; and 2) to determine whether host resistance affects the evolution of virulence and fungicide resistance.

*Mycosphaerella graminicola* (Fückel) Schroeter (anamorph *Septoria tritici*) is the causal agent of septoria leaf blotch on wheat [15], [16]. The pathogen has a global distribution and can cause up to 40% yield loss in many areas of the world [17]. The life cycle of the pathogen involves both sexual and asexual reproduction [15], [16]. Wind-dispersed ascospores produced by the teleomorph contribute significantly both to initiation and further development of disease epidemics [18] and are likely to be one of the main mechanisms contributing to long distance gene flow [19] and host adaptation [20]. Genetic variation in *M. graminicola* populations is high [21] as a result of frequent sexual recombination [18], [20], high gene flow [22] and large effective population size [22]. Results from experimental evolution and population genetic studies indicate that the genetic structure of the pathogen can change significantly over a single growing season in response to host selection [23], while local adaptation leads to significant population differentiation for virulence [24], fungicide resistance [25] and temperature sensitivity [26].

Though both quantitative and qualitative resistances have been identified in wheat hosts, the majority of resistant cultivars used in commercial production display quantitative resistance (QR) to the pathogen [27], [28]. QR is believed to be more durable because natural selection is thought to operate more slowly on quantitative traits. Unlike qualitative resistance (also called major gene resistance), QR is thought to be mediated by several genes each contributing small but additive effects to the overall host resistance [29]. It is thought that mechanisms underlying QR in plants involve preformed, constitutive, physical and chemical barriers, Pathogen-Associated Molecular Pattern (PAMP)-triggered responses [5] and pathogen life-history traits [30]. Interactions of these mechanisms hinder the growth, penetration, reproduction and transmission of a pathogen. QR in plants slows down but does not prevent epidemics, thus effective disease control may require supplementary applications of fungicides.

Triazoles represent a major category of fungicides used widely in agriculture and medicine. This group of fungicides inhibits cytochrome P450 sterol 14 alpha-demethylase, an enzyme required for the biosynthesis of ergosterol in many fungi [31]. Resistance to triazoles is thought to be polygenic [32] and mediated by several mechanisms including mutations in the target protein gene *CYP51* and increased active efflux by ABC transporters [33], [34], [35], [36]. Cyproconazole is a triazole fungicide that has been used for many years to control *M. graminicola* [32].

## Materials and Methods

### Ethics Statements

We confirm that no specific permits were required for the described field study and to collect samples from these locations. We further confirm that the locations were not privately-owned or protected in any way and the field study did not involve endangered or protected species.

### Pathogen populations

Five *M. graminicola* populations sampled from four geographical locations, including one population each from Australia, Israel and Switzerland and two populations from Oregon, USA [21], [26], were used for this study. The Australian population (AUS) was collected near Wagga Wagga in 2001. The Israel population (ISR) was collected near Nahal Oz in 1992 and the Swiss population (SWI) was sampled near Winterthur, kanton Zurich in 1999. The two USA populations (ORER and ORES) were collected on the same day in 1990 from a field planted to the partially resistant cultivar Madsen and the highly susceptible cultivar Stephens, respectively. The fungal isolates were stored in silica gel at −80°C after they were isolated from infected leaves.

Isolates in each population were genotyped using restriction fragment length polymorphisms (RFLPs) and DNA fingerprints. The genotype data were published earlier [21]. Only isolates with a distinct multi-locus RFLP haplotype and DNA fingerprint were chosen for virulence and fungicide resistance tests. A total of 141 genetically distinct isolates were included in the experiment. Each population was represented by 25–30 isolates.

### Measurement of cyproconazole tolerance

*M. graminicola* isolates retrieved from silica gel long-term storage were grown on potato dextrose agar (PDA) amended with 50 mg/L kanamycin and placed at 18°C for seven days. Blastospores formed on these plates were transferred into 50 mL Falcon tubes containing 30 ml yeast sucrose broth (YSB) supplemented with 50 mg/L kanamycin. The tubes were placed at 18°C at 140 rpm for seven days. Spore concentrations for each isolate were determined on the day of inoculation using a haemocytometer and adjusted to 200 spores per mL. 500 μL of the calibrated spore suspension was inoculated onto a PDA plate containing 0.1 ppm cyproconazole while another 500 μL of the spore suspension was inoculated onto a PDA plate without cyproconazole. Our preliminary experiments showed that 0.1 ppm provided the best resolution with the least experimental error. Many isolates did not grow when we used higher concentrations while growth rates of many isolates did not change when we used lower concentrations. Five isolates, one from each of the five populations, were replicated ten times. All other isolates were replicated twice. The inoculated plates were kept at 18°C and colonies on the plates were recorded with a digital camera five days after inoculation. All inoculations and photographs were made by the same person during a single day.

Colony sizes were measured with the image analysis software Assess 2.0 [37]. Cyproconazole tolerance for each isolate was determined by calculating the relative colony size with and without the fungicide, as described previously [24], [25]. Colony sizes were calculated as the average value for the ∼20–70 colonies formed on each plate. Only colonies that clearly developed from single spores were used for the analysis. Fused colonies originating from two or more spores were excluded from the analysis.

### Measurement of virulence

Five 10 cm plastic pots were filled with Ricoter garden soil (Ricoter Erdaufbereitung AG, Switzerland) and sown with ten seeds each of either wheat cultivar Toronit or Greina. Cultivar Toronit was classified as moderately resistant to *M. graminicola* while cultivar Greina was classified as susceptible. The plastic pots were placed in a greenhouse for 21 days at 60% relative humidity and 20°C during daytimes and 40% relative humidity and 16°C during nighttimes. Seedlings were supplemented with 50 kLux florescent light to provide 16 h day-lengths.

*M. graminicola* isolates retrieved from long-term storage were placed on yeast maltose agar plates amended with 50 mg/L kanamycin and kept at 20°C for seven days. Blastospores formed on these plates were transferred into sterile flasks containing 50 ml YSB supplemented with 50 mg/L kanamycin. The inoculated flasks were placed at 20°C with continuous shaking for a week. Spore suspensions were calibrated to a concentration of 5×10^6^ spores per ml on the day of inoculation using a haemocytometer.

Inoculations were made at 21 days after sowing, at approximately growth stage 11 [38]. Seedlings in each pot were thinned to the five most uniform ones and inoculated with 50 ml of the calibrated spore suspension. Leaves of both cultivars were inoculated until run-off with 50 ml of the spore suspension using a semi-automatic sprayer. The inoculated seedlings were placed at 100% relative humidity and 21°C for two days in greenhouse chambers. New plant leaves formed after the inoculation were removed at three day intervals. At 22 days after the inoculation an average of 8–10 leaves was collected from each isolate-cultivar combination. These leaves were mounted onto blue paper sheets and their images were digitized. Percentage Leaf Area Covered by Pycnidia (PLACP) and Percentage Leaf Area Covered by Lesions (PLACL) were measured with the image analysis software Assess 2.0 [37]. All inoculations and all virulence assessments were made during a single day to minimize environmental variance among treatments.

### Data analysis

Frequencies of cyproconazole resistance (natural logarithm transformed) and both PLACL and PLACP (square root transformed) in the fungal isolates were grouped using a binning approach and each group was labelled with the mid-point value of the lower and upper boundaries of the corresponding bins. Analyses of variance for cyproconazole tolerance, PLACL and PLACP were performed using the general linear model procedure implemented in SAS [39]. The raw data were leftward skewed. After applying a natural logarithm transformation to cyproconazole tolerance and a square root transformation to the PLACL and PLACP datasets, the transformed data showed a more even distribution than the non-transformed data, so the transformed data were used in the ANOVA. Least significant differences [40] were used to compare cyproconazole tolerance, PLACL and PLACP among populations sampled from different regions and hosts.

The environmental variance of cyproconazole tolerance, PLACL and PLACP in each population was estimated using the among-replicate variance [41], [42]. In common garden experiments with asexually reproducing species, any variance among replicates can be attributed to environmental effects because individuals in different replicates have the same genotype (i.e. they are identical clones). Therefore, variance among replicates in this case is equivalent to the environmental variance of cyproconazole tolerance, PLACL and PLACP (for details see [24], [41], [42]). Our earlier analyses [24] indicated that the environmental variance estimated using the large number of isolates with two replicates was not significantly different from the variance estimated using the limited number of isolates with 10 replicates. Therefore, we included all isolates in the analysis of environmental variance. Genetic variance in each *M. graminicola* population was estimated by subtracting the environmental variance from the phenotypic variance in the corresponding population. The association between virulence and fungicide tolerance in the pathogen populations was evaluated by simple linear correlation using transformed data [39].

## Results

### Variation in PLACL, PLACP and cyproconazole tolerance in the *Mycosphaerella graminicola* populations

PLACLs ranged from 1–90% with an average (95% confidence interval) of 26% (±3%) on the moderately resistant cultivar Toronit and from 1–90% with an average (95% confidence interval) of 39% (±4%) on the susceptible cultivar Greina, while PLACPs ranged from 0–50% with an average (95% confidence interval) of 9% (±2%) on Toronit and from 0–71% with an average (95% confidence interval) of 24% (±3%) on Greina, respectively.

Frequency distributions of cyproconazole tolerance (natural logarithm transformed) and both PLACL and PLACP (square root transformed) were visualized by grouping isolates into 11, 10 and 13 bins differing by 0.56, 1.0 and 0.70 units, respectively. Our analyses revealed that these bin allocations yielded the optimum distribution to display the trait frequency, with enough strains occupying each bin to calculate and display a frequency with equal spacing between bin means.

**Figure 1:**
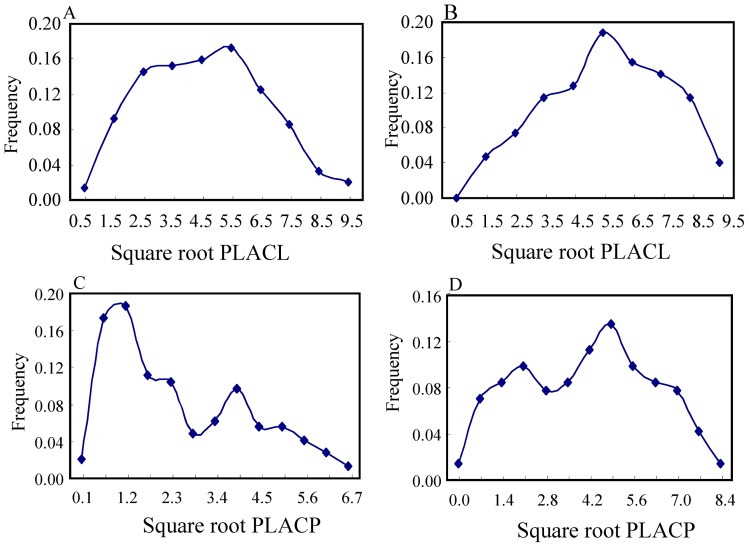
Frequency distribution of Percentage Leaf Area Covered by Lesions (PLACL) and Percentage Leaf Area Covered by Pycnidia (PLACP) in 141 isolates of *Mycosphaerella graminicola* evaluated on two Swiss wheat cultivars. Both PLACL and PLACP were square root transformed and labelled using the mid-point values of the corresponding bins: A) PLACL on Toronit; B) PLACL on Greina: C) PLACP on Toronit; and D) PLACP on Greina.

The square root transformed distributions of PLACL were unimodal and symmetric, peaking at the post-transformation level of 6 (Fig. 1A, 1B) for both cultivars. The square root transformation of PLACP displayed a bimodal distribution on both cultivars with one major peak and one minor peak (Fig. 1C, 1D). On Toronit, the majority of isolates displayed a lower level of virulence, forming a major peak at the post-transformation level of 1 and a minor peak at the post-transformation level of 4. On Greina, the major peak was shifted to the right at the post-transformation level of 5 while the minor peak was shifted to the left at the post-transformation level of 2.

**Figure 2:**
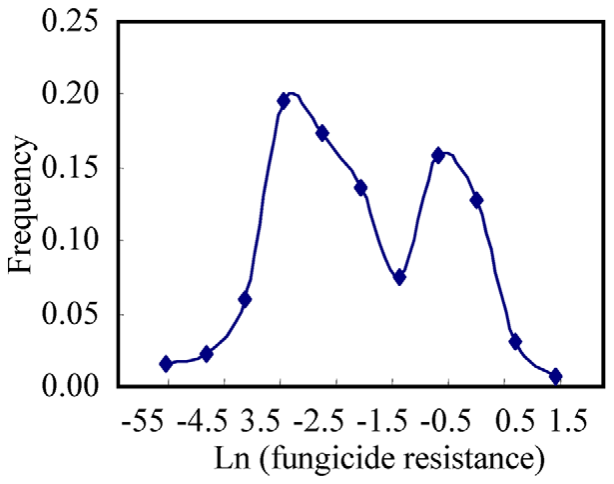
Frequency distribution of cyproconazole resistance in 141 isolates of *Mycosphaerella graminicola*. Cyproconazole resistance was determined by calculating the relative colony size of an isolate grown on Petri plates with and without the fungicide. Data were natural logarithm transformed and labelled using the mid-point values of the corresponding bins.

20–70 colonies were formed on each plate and there was no systematic difference in the number of colonies formed between the plates supplemented and not supplemented with cyproconazole. Tolerance to cyproconazole ranged from 0.01–2.30 with an average of 0.34. Six out of 141 (4%) isolates grew better in the presence of cyproconazole than without the fungicide, resulting in values of fungicide tolerance larger than 1. Like PLACP, the natural logarithm of tolerance to cyproconazole within a pathogen population displayed a bimodal distribution with a major peak at the post-transformation level of −2.6 and a minor peak at the post-transformation level of −0.4 (Fig. 2).

### Comparison of cyproconazole tolerance and virulence among *M. graminicola* populations

Isolate and origin of isolate contributed significantly (p<0.0001) to cyproconazole tolerance, PLACL and PLACP. Cultivar also contributed significantly (p<0.0001) to PLACL and PLACP. Least significant difference analyses showed that the population from Switzerland displayed the highest levels of cyproconazole tolerance and virulence while the population from Australia displayed the lowest levels of cyproconazole tolerance and virulence (Table 1). The population from the resistant host Madsen displayed a higher cyproconazole tolerance and virulence than the population from the susceptible host Stephens (Table 1). All six isolates growing better in the presence of cyproconazole had a Swiss origin.

**Table 1:** LSD test for differences in cyproconazole resistance and virulence among the five *Mycosphaerella graminicola* populations sampled from Australia, Israel, Switzerland and USA.

Populations	Cyproconazole resistance	PLACL (%)^1^	PLACP (%)^2^
SWI	0.82 a^3^	37.8 a	20.7 a
ORE. R	0.29 b	35.1 a	17.3 a
ISR	0.26 bc	29.3 a	16.9 ab
ORE. S	0.16 c	33.3 a	13.2 bc
AUS	0.15 c	20.5 b	7.5 c

1 Percentage Leaf Area Covered by Lesions.

2 Percentage Leaf Area Covered by Pycnidia.

3 Values followed by different letters are significantly different at P≤0.05.

### Correlation between cyproconazole tolerance and virulence of *M. graminicola* isolates

**Figure 3:**
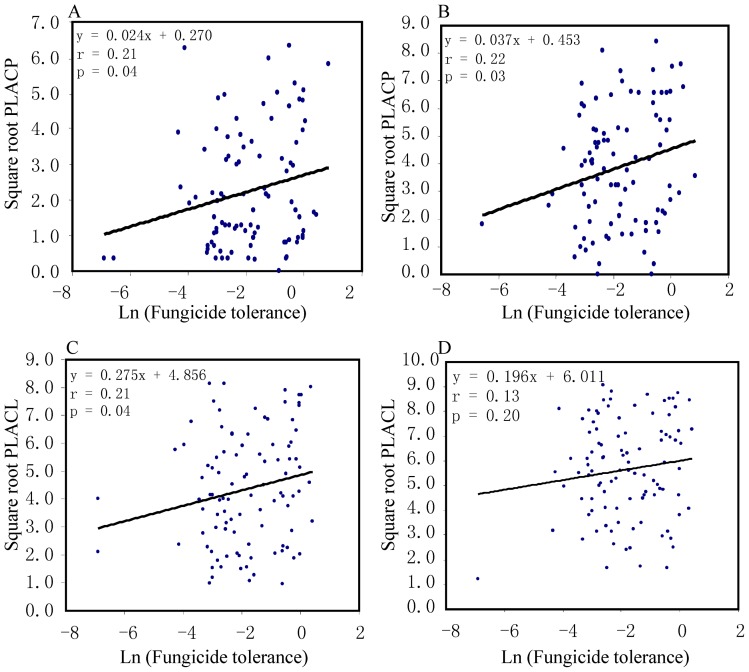
Correlations between cyproconazole resistance and two measures of virulence in 141 isolates of *Mycosphaerella graminicola* evaluated on two Swiss wheat cultivars. Cyproconazole resistance was determined by calculating the relative colony size of an isolate grown on Petri plates with and without the fungicide: A) Percentage Leaf Area Covered by Pycnidia (PLACP) on Toronit; B) Percentage Leaf Area Covered by Pycnidia (PLACP) on Greina; C) Percentage Leaf Area Covered by Lesions (PLACL) on Toronit; and D) Percentage Leaf Area Covered by Lesions (PLACL) on Greina.

**Figure 4:**
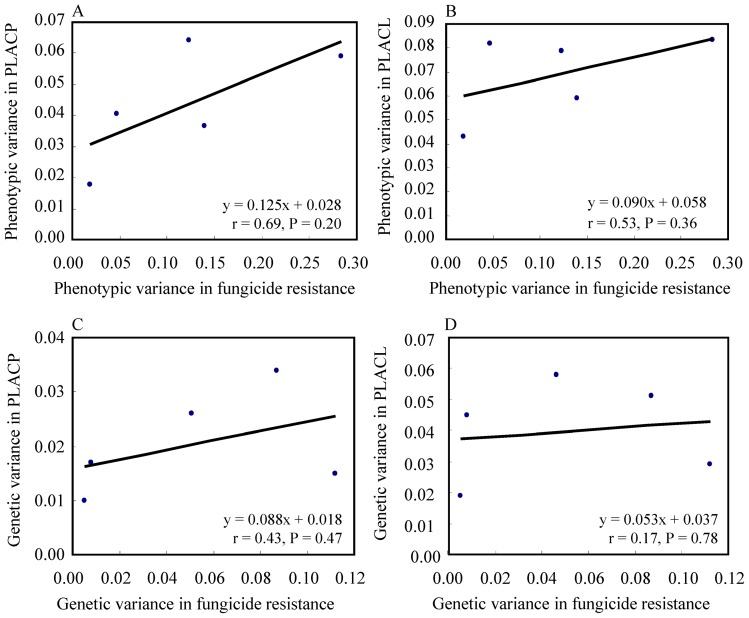
Correlation between variation in cyproconazole resistance and variation in two measures of virulence for five populations of *Mycosphaerella graminicola*. Cyproconazole resistance was determined by calculating the relative colony size of an isolate grown on Petri plates with and without the fungicide. Correlation was estimated at the population level: A) phenotypic variation in Percentage Leaf Area Covered by Pycnidia (PLACP); B) phenotypic variation in Percentage Leaf Area Covered by Lesions (PLACL); C) genetic variation in Percentage Leaf Area Covered by Pycnidia (PLACP); and D) genetic variation in Percentage Leaf Area Covered by Lesions (PLACL).

**Figure 5:**
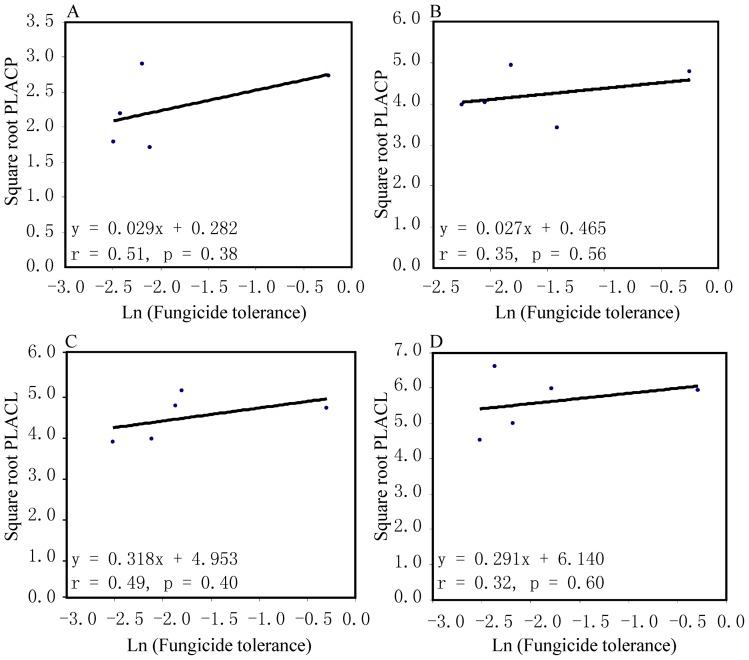
Correlation between population mean in cyproconazole resistance and two measures of virulence in *Mycosphaerella graminicola*. Cyproconazole resistance was determined by calculating the relative colony size of an isolate grown on Petri plates with and without the fungicide: A) Percentage Leaf Area Covered by Pycnidia (PLACP) on Toronit; B) Percentage Leaf Area Covered by Pycnidia (PLACP) on Greina; C) Percentage Leaf Area Covered by Lesions (PLACL) on Toronit; and D) Percentage Leaf Area Covered by Lesions (PLACL) on Greina.

There were significant correlations between cyproconazole tolerance and PLACP on both Toronit (r_91_  = 0.21, p = 0.04 Fig. 3A) and Greina (r_91_  = 0.22, p = 0.03, Fig. 3B). The correlation coefficient between cyproconazole tolerance and PLACL on Toronit was positive and significant (r_97_  = 0.21, p = 0.04, Fig. 3C). Though the correlation coefficient between cyproconazole tolerance and PLACL on Greina was also positive, it was not significant (r_98_  = 0.13, p = 0.20, Fig. 3D), Variances and population means of cyproconazole tolerance and virulence were also positively associated, but none of them were significant (Fig. 4 & 5).

## Discussion

We assayed virulence and tolerance to a triazole fungicide in a large collection of *M. graminicola* isolates sampled across several host genotypes and geographic locations. We found positive correlations between virulence and fungicide tolerance (Fig. 3), suggesting an association between these two quantitative traits. In an earlier experiment conducted in Oregon, USA, Cowger and Mundt [43] also found that *M. graminicola* isolates from cultivars treated with the protectant fungicide chlorothalonil were more aggressive than isolates sampled from the same cultivars in nearby, untreated fields. It is not clear whether the positive correlation between virulence and fungicide tolerance observed in pathogens sampled from agricultural ecosystems will also be found in pathogens sampled from natural ecosystems. Additional studies with other agricultural pathogens and with pathogens collected from natural systems will be needed to determine the generality of these findings. The lack of significant correlations between variances and means in virulence and cyproconazole tolerance at the population level could be due to the small number of data points available for this comparison. Because only five populations originating from four geographic locations were included in this study, associations would need to be very high (r>0.89) to detect a significant correlation with such a small number of data points.

Local adaptation and population differentiation can affect the estimate of association between ecological characters [44], [45]. Extensive utilization of fungicides and quantitative resistance in some regions may result in both high virulence and high fungicide tolerance. In *M. graminicola,* we found that the Australian population had the lowest overall virulence and cyproconazole tolerance while the Swiss population had the highest overall virulence and cyproconazole tolerance [25], consistent with significant local adaptation and a high level of population differentiation for the two characters. To eliminate the possible effect of this population structure on our conclusions, the association between fungicide tolerance and virulence was further evaluated using a randomisation procedure [46]. The fungicide and virulence datasets in the Switzerland and Australia populations were randomized and then added to the original dataset (without randomization) of the other three populations to calculate correlation coefficients. The process was repeated 10000 times. Results from the randomization analysis revealed that the observation of a positive association between the two traits could not be attributed to local adaptation or population differentiation (data not shown).

We also found significantly higher PLACL, PLACP and cyproconazole tolerance in the pathogen population sampled from the resistant wheat cultivar Madsen than the susceptible cultivar Stephens (Table 1). These two pathogen populations were sampled from the same field at the same point in time and therefore most likely originated from the same source population. Because no triazole fungicides were applied to this field, and fungicide use was rare in this region, we do not believe that the difference in triazole tolerance between the *M. graminicola* populations from the resistant and susceptible hosts is due to selection for fungicide tolerance. This interpretation is supported by the lack of *CYP51* sequence variation among isolates from the two hosts [25]. Instead, we hypothesize that the resistant host selected for higher pathogen virulence, which in turn was linked to or had secondary functions related to triazole tolerance (see below for details). Resistant hosts selecting for higher pathogen virulence has already been predicted theoretically [47], [48] and reported from experiments [49], [50]. Because quantitative host resistance decreases pathogen growth rate, pathogens can compensate for lower growth rates by evolving towards an increasing competitive ability, which in turn can result in increased virulence [46].

Both linkage (i.e. hitch-hiking) and pleiotropic effects could lead to a positive association between virulence and fungicide tolerance in pathogens, but hitch-hiking is unlikely to be the cause in this case. First, hitch-hiking refers to the process through which an allele increases in frequency because it is linked to an allele that is under positive selection [51], [52]. Cyproconazole tolerance, PLACL and PLACP are quantitative traits that display continuous variation within populations (Figs. 1–2). It is possible that each of these traits is affected by many minor genes, but it is unlikely that all or most of the genes contributing to the increase of cyproconazole tolerance are closely linked to the genes governing the increase of PLACL or PLACP. Second, recombination rate plays a key role in determining the degree of hitch-hiking [53]. Hitch-hiking effects are expected to be lower in populations with high recombination rates. *M. graminicola* populations display a high degree of sexual recombination both during and between growing seasons [18], [20] and the populations included in this study were at gametic equilibrium [21], [22]. Thus, even if there were close linkage between the genes encoding cyproconazole tolerance and virulence, the high recombination rate observed in populations of *M. graminicola* would lead to a rapid decay in disequilibrium.

We hypothesize that the observed correlation is due to pleiotropic effects of genes that affect both virulence and cyproconazole tolerance. Host defense systems usually involve the production of compounds that have lethal or inhibitory effects on the penetration, survival and reproduction of pathogens [54]. These defense-related compounds may share some structural or functional characteristics with synthetic antimicrobials. Pathogen strains having the ability to detoxify the compounds produced by resistant hosts may also have the ability to detoxify synthetic antimicrobial compounds, leading to a simultaneous increase in virulence and antimicrobial resistance. This detoxification process could involve mechanisms such as reducing the entry of natural and synthetic compounds into pathogen cells through the action of efflux pumps located in the cytoplasmic membrane. It has been reported that some efflux pumps, such as ABC transporters and MgrA protein, have the ability to transport a broad range of structurally unrelated compounds during pathogen infection, therefore affecting both virulence and antimicrobial resistance, in many plant and human pathogens [55], [56], [57], [58], [59].

The positive correlation between virulence and cyproconazole tolerance could also be due to pathogen metabolites that can destroy or modify the structures and functions of both natural and synthetic antimicrobials. An example of such a defense metabolite is melanin. Melanin is composed of dark-brown or black pigments formed by the oxidative polymerization of phenolic compounds and can be produced by a broad array of plant and human pathogens [60], [61], [62]. It has been documented that melanin can increase antimicrobial resistance (see [63] for review) by reducing the susceptibility of melanized cells to antimicrobials [64], [65], [66] and increase virulence by interfering with numerous host defense mechanisms [67], [68], [69], [70], [71], [72] in many human pathogens. In *M. graminicola* strain IPO323, Mehrabi et al. [59] found that disruption of *MgSlt2* in *M. graminicola* led to a loss of melanization on potato dextrose agar, a loss of virulence and increased sensitivity to several fungicides including cyproconazole. Choi and Goodwin [60] also found that the velvet gene *MVE1* is involved in the synthesis of melanin in *M. graminicola*. *MVE1* mutants produced significantly less melanin. In *Fusarium graminearum,* deletion of the homologous velvet gene (*FgVEA*) reduced virulence and increased fungicide sensitivity [73].

The finding of a positive association between pathogen virulence and tolerance to synthetic antimicrobials coupled with the knowledge that resistant plant hosts can select for higher pathogen virulence has many implications for sustainable disease management in agroecosystems. It suggests that one unforeseen consequence of widespread deployment of quantitatively resistant cultivars or intensive application of synthetic antimicrobials might be selection for a higher basal level of antimicrobial resistance and enhanced virulence in pathogen populations, which would pose a greater threat to agricultural production. In this case, more dynamic disease management programs that incorporate more rapid spatial and temporal turnover of host resistance or synthetic antimicrobials may be important for sustainable disease control [74]. More rapid spatial and temporal turnover of host resistance or antimicrobials is expected to generate fluctuating selection against pathogens that could prevent the emergence of pathogen individuals and populations with higher virulence and antimicrobial resistance. However, the effectiveness of the proposed strategy for disease management depends largely on the fitness costs associated with virulence or antimicrobial resistance. If there are no fitness costs, then there may be no benefit derived from spatial and temporal deployments of host resistance or antimicrobials.
